# Matrix Metalloproteinases as Biomarkers of Atherosclerotic Plaque Instability

**DOI:** 10.3390/ijms21113946

**Published:** 2020-05-31

**Authors:** Wioletta Olejarz, Dominika Łacheta, Grażyna Kubiak-Tomaszewska

**Affiliations:** 1 Department of Biochemistry and Pharmacogenomics, Faculty of Pharmacy, Medical University of Warsaw, 02-097 Warsaw, Poland; dominika.lacheta@wum.edu.pl (D.Ł.); grazyna.kubiak-tomaszewska@wum.edu.pl (G.K.-T.); 2 Centre for Preclinical Research, Medical University of Warsaw, 02-097 Warsaw, Poland

**Keywords:** matrix metalloproteinases (MMPs), atherosclerosis, biomarkers, doxycycline, statins

## Abstract

Matrix metalloproteinases (MMPs) are a family of zinc-dependent endopeptidases responsible for tissue remodeling and degradation of extracellular matrix (ECM) proteins. MMPs may modulate various cellular and signaling pathways in atherosclerosis responsible for progression and rupture of atherosclerotic plaques. The effect of MMPs polymorphisms and the expression of MMPs in both the atherosclerotic plaque and plasma was shown. They are independent predictors of atherosclerotic plaque instability in stable coronary heart disease (CHD) patients. Increased levels of MMPs in patients with advanced cardiovascular disease (CAD) and acute coronary syndrome (ACS) was associated with future risk of cardiovascular events. These data confirm that MMPs may be biomarkers in plaque instability as they target in potential drug therapies for atherosclerosis. They provide important prognostic information, independent of traditional risk factors, and may turn out to be useful in improving risk stratification.

## 1. Introduction

Matrix metalloproteinases (MMPs) are a family of zinc-dependent endoproteases responsible for tissue remodeling and degradation of extracellular matrix (ECM) proteins [1]. MMPs are secreted by endothelial cells, vascular smooth muscle, fibroblasts, osteoblasts, macrophages, neutrophils, and lymphocytes [2]. MMPs family contain 28 members, 23 are expressed in human tissues and 14 are expressed in veins and arteries. They are classified on the basis of their substrates and the organization of their structural domains [3]. MMPs may be inhibited by tissue and biological and synthetic inhibitors [4]. Endogenous tissue inhibitors of metalloproteinases (TIMPs) are widely distributed in many tissues and organs. In the case of biological inhibitors, doxycycline (an antibiotic) remains the single MMPs inhibitor that was approved by Food and Drug Administration (FDA) for clinical use [5]. Importantly, statins exert pleiotropic effects in vivo, including influence on signaling mechanisms that leads to MMPs inhibition. [6]. MMPs play important role in maintaining vein wall structure and function, but on the other hand, in adverse cardiovascular remodeling, atherosclerotic plaque formation and plaque instability [7,8]. Serum elevation of MMP-2, ADAMTS-1, and ADAMTS-7 is correlated with the initial stages of chronic venous disease (CVD), whereas the serum elevation of MMP-1, MMP-8, MMP-9, NGAL, ADAM-10, ADAM-17, and ADAMTS-4 is particularly involved in skin change complications [9].

Increased activity of MMP-7 and MMP-9 was observed in unstable plaques, the highest tissue expression of MMP-9 was found in plaques of lipid type compared with plaques of necrotic and inflammatory-erosive types [10]. In addition, MMP-7 could contribute to plaque instability in carotid atherosclerosis, potentially involving macrophage-related mechanisms [11]. MMP-9 is a strong independent predictor of atherosclerotic plaque instability in stable coronary heart disease (CHD) patients, where MMP-9 levels are positively associated with the size of the necrotic core of coronary atherosclerotic plaques [12]. It was shown that serum MMP-9 and the MMP-9/TIMP-1 molar ratio may be valuable in acute coronary syndrome (ACS) diagnosis and prognosis. MMP-9 activation in serum was associated with poor cardiovascular outcome [13]. Tan et al. investigated associations of MMP-9 and monocyte chemoattractant protein-1 (MCP-1) concentrations with the severity of carotid atherosclerosis, based on measurements of carotid plaque and intima–media thickness (IMT). Elevated serum MMP-9 concentration was independently associated with high total carotid artery plaque score, plaque instability, and large IMT value [14]. Importantly, using selective MMP-9 inhibitors may provide new perspective to intervene on ECM remodeling in humans [15]. These data confirm that MMPs may be biomarkers and have been proposed as potential therapeutic targets in cardiovascular diseases [16,17,18].

## 2. MMPs Structure and Tissue Distribution

**Figure 1:**
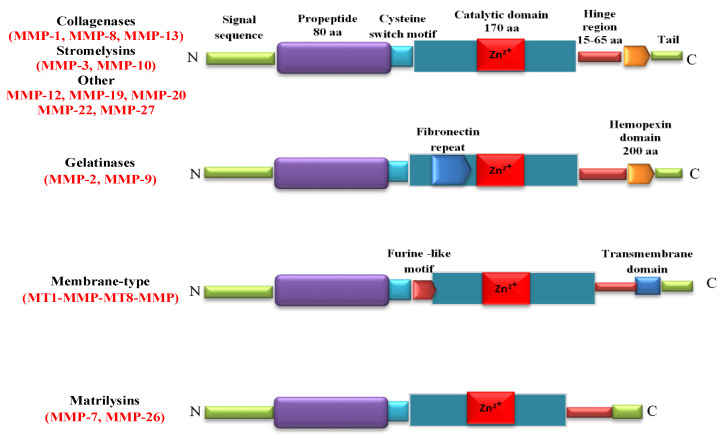
Structure of matrix metalloproteinases (MMPs). Catalytic domain contains Zn^2+^ in the active site. Signal sequence and prodomain are removed during the proteolytic activation of pro-MMPs. Cysteine-rich switch is essential for the activation of MMPs. The hinge region serves as a linker between the catalytic domain and C-terminal domain.

Matrix metalloproteinases (MMPs) are Zn^2+^ endopeptidases. They contain about 80 types of amino acids, a catalytic metalloproteinase domain (containing about 170 amino acids), a binding type of the special peptide or hinge-shaped region of variable length and a hemopexin stationary domain, with about 200 amino acids [19]. A definition of MMPs is based on their substrate specificity, structural organization, and cellular location. From a chemical point of view, the MMPs include collagenases (MMP-1/collagenase-1, MMP-8/collagenase-2, and MMP-13/collagenase-3), stromelysins (MMP-3/stromelysin-1, MMP-10/stromelysin-2, and MMP-11/stromelysin-3), gelatinases (MMP-2/gelatinase-A and MMP-9/gelatinase-B), membrane-type MMPs such as transmembrane MMP-14, MMP-15, MMP-16, MMP-24, membrane-anchored MMP-17, and MMP-25, and matrilysins (MMP-7/matrilysin-1 and MMP-26/matrilysin-2 [20] (Figure 1).

The MMP-1, MMP-2, MMP-3, MMP-7, and MMP-9 expression was found in endothelial cells and vascular smooth muscle cells (VSMCs), while MMP-12 showed expression in VSMCs and fibroblasts [21]. MMP-1, MMP-3, MMP-7, MMP-9, MMP-13, a membrane-type (MT) MMPs, MT-MMP1, and MT-MMP3 were found in the vascular wall [22]. Indeed leukocytes and dermal fibroblasts are key sources of MMP-2, whereas platelets are source of MMP-1, MMP-2, MMP-3, and MMP-14 [23] (Table 1).

## 3. Extracellular Vesicles as MMPs Carriers

Extracellular vesicles (EVs) as structures secreted by cells by the paracrine route include exosomes (diameter range: 30–100 nm), apoptotic bodies (diameter range: 0.1–1 μm), microvesicles (MVs, diameter range: > 1 μm), and large oncosomes (diameter range: 1–10 μm). They are released from cells as a result of fusion of the endosome with the plasma membrane. EVs participate in intercellular communication [24,25,26]. The composition of the microvesicles depends on the cell that secretes these structures. It is determined by a set of sorting proteins [27]. The mechanisms of transport of MMPs to extracellular vesicles are not fully understood. Sinha’s and Clark’s researches indicated that cortactin, as a regulator of matrix metalloproteinase secretion, was involved in this process [28,29]. In addition, the role of Rabi GTPases and kinesins in MMPs exposure to stem cell surface and packaging of these proteins into exosomes has been demonstrated [30,31]. In turn, the location of MMP-14 in exosomes depends on vesicle-associated membrane protein 3 (VAMP-3) [32].

EVs are involved in the secretion of MMPs into the intercellular space. As carriers of numerous proteins, including matrix metalloproteinases, extracellular matrix metalloproteinase inducer (EMMPRIN), and tissue inhibitors of metalloproteinases (TIMPs), they can affect the reconstruction of the intercellular matrix, resulting in, among others atherosclerotic plaques destabilization [33]. Moreover, it has been shown that EVs not only transport MMPs but also participate in their activation. Studies by Bobryshev et al. have shown that at preatherosclerotic disease stage, the secretion of MMP-enriched EVs from arterial smooth muscle cells is significantly greater in the athero-prone areas of the human aorta than in the athero-resistant areas [34]. Research by Laghezza et al. showed the involvement of EVs in the proteolytic activation and secretion of MMP-9. Extracellular vesicles secreted by endothelial cells and platelets, as MMP-9 carriers, participate in the regulation of atherosclerotic plaque neovascularization. Angiogenesis is promoted at low levels of endothelial-derived microvesicles containing active MMP-2 and MMP-9, while high levels of these MVs inhibit plaque angiogenesis which reduces the risk of rupture [35]. In addition, Lozito et al. demonstrated that EVs secreted from endothelial cells are involved in the activation of MMP-2 initiating remodeling of the vascular matrix [36,37]. MMP-2 present in EC-derived vesicles also participates in neovascularization, promoting capillary structure formation and plaque rupture [25,38]. It is known that MT1-MMP present in exosomes secreted by fibroblasts participates in the pro-MMP2 activation process [39]. In turn, studies in ApoE (-/-) mice have showed that olmesartan significantly limit the formation of atherosclerotic lesions in the aorta by reducing the activity of MMP-2 [40].

MMP-rich microvesicles are effective modulators of weakening of the fibrous cap. Neutrophil-derived MVs enriched in MMP-9, metalloproteinase domain-containing proteins 10 (ADAM-10), and -17 (ADAM-17), as well as matrix-derived MVs rich in MMP-2 and endothelial MMP-10-enriched MVs, play a huge role in this process [41]. In addition, exosomes secreted by various cells, including macrophages, transporting MMP-2, MMP-3, MMP-9, and MMP-13, simultaneously contribute to vascular calcification. This increases plaque susceptibility to rupture, leading to life-threatening cardiovascular events such as myocardial infarction [35,42,43,44]. Studies in patients with chronic kidney disease have shown that under mineral imbalance, there is an increased secretion of MMP-2-rich exosomes from vascular smooth muscle cells, which converts the exosomes into loci to endothelial calcification [45]. Chondrocyte-derived MVs containing MMP-3 participate in the calcification process of extracellular vesicles in the fibrous collagen of the extracellular matrix of cardiovascular tissues. Their secretion is induced by phosphate through extracellular kinases regulated by the Erk1/2 signal pathway [33].

Exosomes may serve as biomarkers for the development of atherosclerosis, providing potential roles for diagnosis and treatment [46]. Quantitative analysis of MMP-rich extracellular vesicles (especially those containing MMP-2 and -9) can be used as a parameter to assess calcification and plaque neovascularization in monitoring the clinical course of cardiovascular diseases. MVs analysis also seems to be helpful in identifying patients at risk of cardiovascular disease. MVs can also be a therapeutic target for preventing and controlling the development of atherosclerosis, including progression of plaque formation and its instability [40,47].

## 4. Biological Inhibitors of MMPs

Among tissue inhibitors of MMPs (TIMPs), such as tetracyclines, chemically-synthesized small-molecular weight MMP inhibitors and inhibitory antibodies, only doxycycline, which was approved by FDA, has been evaluated extensively in patients [48]. In a TIPTOP study, doxycycline decreased infarction size, and showed improvement of cardiac contractility characteristic in patients with acute ST-segment elevation myocardial infarction (STEMI) and left ventricular (LV) dysfunction [49]. Additionally, in another clinical trial, short-term treatment with doxycycline caused reduction of MMP-2 activity in coronary bypass patients [50]. It was shown that doxycycline attenuates heart mechanical dysfunction and reduces inflammation in patients with coronary artery disease (CAD) [51]. In addition to its potential plaque stabilization in acute coronary syndromes properties, doxycycline shows promise in preventing ischemia-reperfusion injury and left ventricular remodeling [52]. Furthermore, clinical study demonstrated that doxycycline therapy led to downregulation of MMPs, dampening cardiac inflammation, and reduction of secondary myocardial infarction (MI) risk in coronary bypass patients [53].

The statins may inhibit expression of MMPs in atherosclerotic plaques by reducing vascular inflammation [54,55]. Atorvastatin inhibits MMP-1, MMP-2, and MMP-9 expression in human retinal pigment epithelial cells [56] and MMP-1, MMP-2, MMP-3, and MMP-9 secretion from rabbit macrophages and cultured rabbit aortic and human saphenous vein VSMCs [57]. Komukai et al. in the EASY-FIT clinical trial indicated that atorvastatin increased fibrous cap thickness and plaque stability and decreased levels of MMP-9 in CAD patients [58]. Further, Liu et al. confirmed that atorvastatin reduced plasma MMP-9 levels and myocardial dysfunction in STEMI [59]. Li et al. demonstrated that simvastatin significantly suppressed LPS-induced MMP-9 release and mRNA expression in a time- and concentration-dependent manner [60]. It was shown that in a rat model of heart failure, pravastatin suppressed the increase in myocardial MMP-2 and MMP-9 activity [61]. In addition, rosuvastatin inhibits the expression of MMP-2 and MMP-9 [62]. Moreover, a recent study reported the efficiency of combined therapy with rosuvastatin and ezetimibe to treat plaque instability and cardiovascular inflammation in CAD patients, by a significant decrease in MMP-9 plasma concentration [63]. Sapienza et al. recently showed that patients with advanced PAD, a pathological condition of hemodynamic instability of the atheromatous plaque, had good results from the association between platelet aggregation inhibitors and statins in terms of patency of the reconstructions [64,65].

## 5. MMPs in Pathogenesis of Atherosclerosis

Atherosclerosis is a chronic inflammatory disease of the vessel wall that is largely driven by an innate immune response [66]. In pathogenesis of atherosclerosis, a pivotal role is played by innate immunity receptors such as toll-like receptors (TLR) and receptors for advanced glycation end products (RAGE) [67]. TLR and RAGE mediate in macrophages and leukocyte recruitment and are significantly involved in the initiation and progression of atherosclerosis [68]. This process is characterized by the accumulation of lipids, smooth muscle cell proliferation, cell apoptosis, necrosis, and fibrosis [69]. MMPs plays a key role in all stages of atherosclerosis through vascular inflammation, endothelial dysfunction, smooth muscle cell migration, vascular calcification, extracellular matrix degradation, and plaque activation and destabilization [8,16,70]. The expression of MMPs is controlled by different microRNA molecules. MicroRNA regulation of extracellular matrix components is crucial in the process of atherosclerotic plaque destabilization [71]. In the artery wall, subendothelial retention of plasma lipoproteins triggers monocyte-derived macrophages and T helper type 1 (Th1) cells to form atherosclerotic plaques. Plaque rupture or endothelial erosion can induce thrombus formation, leading to myocardial infarction or ischemic stroke [72]. Ruptured plaques are characterized by a large lipid-rich core, a thin fibrous cap that contains few smooth muscle cells and many macrophages, angiogenesis, adventitial inflammation, and remodeling. Plaque rupture is the most common cause of coronary thrombosis [73]. It was shown that endothelial-to-mesenchymal transition (EndMT) is a cellular reprogramming mechanism by which endothelial cells acquire a mesenchymal phenotype. EndMT contributes to the initiation and progression of atherosclerotic lesion and plaque destabilization, which further causes acute cardiovascular events [74].

### 5.1. MMPs in Vascular Inflammation and Recruitment of Immune Cells

MMPs participate in the immune response and play a key role in vascular inflammation that is strongly associated with atherosclerosis [75]. Exposure of vascular wall cells to inflammatory mediators produced under chronic inflammation may lead to excessive MMPs activity in the arterial wall resident and recruited cells [70,76]. In response to proinflammatory mediators, monocytes enhance MMPs expression and play a key role in inflammatory cell migration and invasion into the arterial wall [77]. Human monocytes not only express TIMP-1, TIMP-2, MMP-8, MMP-12, and MMP-19 but also activate MAP kinase and NF-κB, and in this way promote the expression of MMP-2, MMP-3, MMP-10, and MMP-14. The ratio between MMPs and their inhibitors (TIMPs) to free active MMPs is critical in pathogenesis of cardiovascular diseases [78]. TIMP-2 plays a protective role in the pathogenesis of atherosclerosis by suppressing MMP-14-dependent monocyte/macrophage accumulation into plaques [79]. MMP-1 expression is augmented in inflammatory and autoimmune diseases through upregulation by inflammatory cytokines such as TNF α (tumor necrosis factor-α) and IL-1 (interleukin-1) [80]. These inflammatory cytokines participate in ROS (reactive oxygen species) production and influence the expression and activity of MMPs. In addition, activation of the PDGFR-β and ERK1/2 atherogenic pathways participates in the production of MMP-1 in human coronary smooth muscle cells (SMCs) stimulated by oxidized low-density lipoproteins (oxLDL) [81].

### 5.2. MMPs in Lipid Accumulation

Migration of macrophages as well as deposition of low-density lipoprotein (LDL) under the endothelium initiate the process of atherosclerosis [82]. Oxidatively modified LDL (oxLDL) are involved in the transformation of macrophages into foam cells and the migration of VSMCs into the intima [83]. Foam cell macrophages, VSMCs, and endothelial cells produce cytokines enhancing the recruitment of inflammatory cells, which in turn attracts VSMCs into the neointima [48]. MMPs and TIMPs are secreted constitutively or after activation by inflammatory response not only by monocytes and macrophages but also by foam cells [84]. oxLDL activates MMP-2 through upregulation of the MT1-MMP expression and stimulation of oxidative radicals production by xanthine/xanthine oxidase [85]. In slow-to-heal wounds increased activity and expression of MMP-1, MMP-8, and MMP-9 have been shown, accompanied with low TIMPs levels [86]. It was shown that MMP-9 can modulate cholesterol metabolism, through MMP-9-plasma secreted phospholipase A_2_ axis [87].

### 5.3. MMPs in Endothelial Dysfunction

MMPs have been found to be involved in vascular wall remodeling and atherosclerosis development through inflammatory activation and endothelium dysfunction [88]. The dysfunction of endothelium is characterized by proinflammatory and prothrombic state; the action of the endothelium is shifted toward reduced vasodilation, and the phenotype changes from antiadhesive to proadhesive [89]. Damage of endothelial junctions results in enhanced endothelial permeability, which facilitates infiltration of various inflammatory mediators. Activation of endothelial MMP-2 can induce endothelial dysfunction and disintegrity [90]. Recruited vascular wall cells can remodel the surrounding extracellular matrix through MMPs that affect migration, proliferation, and apoptosis of endothelial cells and VSMCs [91].

### 5.4. MMPs in Migration of VSMCs

MMPs contribute to intimal thickening and vessel wall remodeling in atherosclerosis by promoting migration and proliferation of VSMCs [92,93]. MMPs degrade ECM and facilitate VSMCs migration into the intima. Studies have shown that MMP-2 is involved in these alterations of VSMCs behaviors. IL-1β, cytokine responsible for VSMCs migration, activates MMP-2 synthesis [94]. MMP-2 plays a key role in ox-LDL-induced activating VSMCs proliferation through various pathways [95]. TNF-α promotes VSMCs migration through MMP-9 upregulation [96]. Enhanced expression of MMP-9 in VSMCs can be caused by angiotensin II (Ang II) that participates in atherosclerosis by NF-κB pathways and angiotensin type 1 receptor [97]. Moreover, MMP-9 stimulates proliferation of VSMCs by regulating cell adhesion and cadherin association [98]. Increased serum MMP-10 levels were connected with increased carotid intima–media thickness, atherosclerotic plaques, and inflammatory markers in patients with preclinical atherosclerosis [99]. Activity of certain variant of the MMP-3 promoter was correlated with progression of luminal narrowing of coronary arteries [100,101].

### 5.5. MMPs in Plaque Neovascularization

Remodeling of extracellular matrix and vascular basement membrane allow to form new blood vessels. Studies have shown that MMP-9 mobilizes vascular endothelial growth factor (VEGF) from the ECM, which can contribute to plaque neovascularization. The increase of VEGF bioavailability results from the ECM proteolysis. [102]. Hypoxia and inflammation in the lesion can induce plaque neovascularization, which leads to plaque rupture [103]. In hypoxia, the direct target of injury are endothelial cells. Hypoxia induces cell proliferation in the vascular wall and results in increased MMP-9 expression in VSMCs [104]. These cells enhance MMPs activity in hypoxic areas. The interaction of macrophages with VSMCs influences neovascularization and destabilization of plaque because of enhanced MMP-1 and MMP-9 production [105]. In neovascularization, MMPs play a crucial role because of their participation in penetration of the ECM and tissue remodeling. The cross-talk between VSMCs and macrophages augments synthesis of angiogenic factors. Both cells produce not only VEGF and IL-1β (cytokine, stimulating the secretion of VEGF) but also MMPs responsible for degradation of ECM components, resulting in penetration and reshaping of connective tissue.

### 5.6. MMPs in Plaque Calcification

MMPs were also demonstrated to provoke vascular calcification [106]. Vascular calcification is determined as an attribute of advanced atherosclerotic plaques. The plasma level of MMP-7 is positively associated with carotid calcification [107]. Additionally, MMP-2 contributes to the calcification of SMCs, participating in the formation of vascular calcified lesions. MMP-2 deficiency in ApoE-/- mice related to suppressed calcium deposition in aorta-derived cultured SMCs [108]. In ApoE-/- mice with advanced atherosclerotic lesion, chondrocyte-like cells were found and several bone-related proteins were expressed [109]. Platelets contain and release MMPs within vascular injury [110]. MMP-2 participates in thrombogenesis, cleaving platelet proteinase-activated receptor 1 (PAR1), and stimulating the aggregation of platelets [111]. Purroy et al. research showed low MMP-10 expression, while significantly reducing the area of atherosclerosis and their calcification in ApoE-/- Mmp10 -/- mice. Furthermore, studies in subjects with subclinical atherosclerosis showed a correlation between MMP-10 serum activity and the degree of coronary calcification [112].

### 5.7. MMPs in Plaque Activation and Destabilization

Studies indicate the involvement of MMPs in atherosclerotic plaque initiation, progression, and plaque rupture [113]. Link between inflammation and plaque vulnerability might provide increased CRP-related MMPs activation [114]. MMP-1 may contribute to the progression of the human atherosclerotic lesions by remodeling of the neointimal extracellular matrix (ECM) [115]. An important role in the pathogenesis of atherosclerosis and plaque rupture may be played by enhanced MMP-1 expression induced by monocyte–endothelial cell interactions [116].

**Figure 2:**
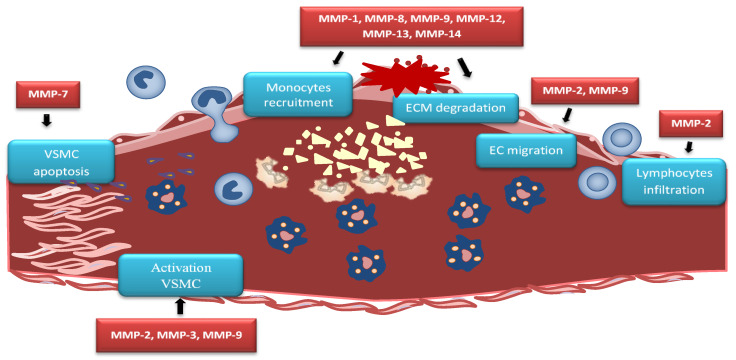
MMPs in atherosclerotic plaque. MMP-2 causes lymphocytes infiltration while MMP-2 and MMP-9 causes endothelial cells (EC) migration. MMP-7 activity leads to vascular smooth muscle cells (VSMC) apoptosis, contributing to plaque instability. Activated VSMCs produce high-mobility group box 1 (HMGB1) which affects the expression of MMP-2, MMP-3, and MMP-9 and enhances plaque rupture. MMP-1, MMP-8, MMP-9, MMP-12, MMP-13, and MMP-14 trigger similar effect by extracellular matrix (ECM) degradation within the fibrous cap that causes plaque rupture.

Advanced atherosclerotic plaque is filled by cell debris, extracellular lipids, and foam cell macrophages, whereas stable plaque is protected from the circulating blood by a fibrous cap. Plaque destabilization can be caused by proteolysis of fibrillar collagens. MMP-1, MMP-8, and MMP-13 show this collagenolytic activity in unstable plaques [117,118]. Studies on human cells infected with influenza A virus showed an increase of MMP-13 expression, which may partly explain the destabilization of atherosclerotic plaques. Analysis of the corresponding changes in the ApoE-deficient mouse model has shown that the increase of MMP-13 expression is due to activation of the p38 mitogen-activated protein kinase signaling pathway [119]. Increased MMP-7 activity can be connected with the apoptosis of VSMCs in the fibrous cap and lesion destabilization [120]. Enhanced MMP-7 activity is involved in the cleavage of N-cadherin, which mediates VSMCs apoptosis, resulting in plaque instability [121]. Vulnerable atherosclerotic plaques are characterized by increased MMPs levels [122]. Activated VSMCs produce high-mobility group box 1 (HMGB1), which affects the expression of MMP-2, MMP-3, and MMP-9 and enhances plaque rupture [123] (Figure 2).

Genetic variants of MMP-9 were found to be associated with different stages of atherosclerosis [124]. Crucial significance of MMP-9 in the growth of atherosclerotic plaque is shown in ApoE-deficient mice [125]. Atherosclerotic plaque formation is associated with hypertension-induced atherosclerosis and increased levels of MMP-9 mRNA [126]. Matrix-degrading activity of MMPs contributes to complications of human atherosclerotic lesions. Studies have shown enhanced expression of MMPs in macrophage-rich areas (around lipid-rich core) suggesting important role of MMPs (derived from macrophages) in developing of vulnerable regions of atherosclerotic plaques [127]. Ablation of MMP-9 contributed to the reduction of atherosclerotic burden and attenuated macrophage infiltration and collagen deposition. In addition, imbalance in the expression of MMP-9/TIMP-1 was perceived in macrophages within the atherosclerotic plaques [128]. A positive correlation between MMP-9 macrophages and plasma lysophosphatidic acid (LPA) concentration was also shown. This is particularly important due to the fact that MMP-9 is derived from macrophages with coronary plaque instability. As demonstrated by Gu et al., LPA acting through the LPA2 receptor can induce MMP-9 by activating the NF-κB pathway [129] (Table 2).

Evidence suggests that neutrophil gelatinase-associated lipocalin (NGAL) may play a crucial role in vascular remodeling and plaque instability during the development of atherosclerosis [130]. NGAL is involved in the regulation of MMP activity. MMPs and NGAL also play a key role in development of arterial aneurysms [131]. The complex formation between NGAL and MMP-9 suggests that NGAL might play a role in progression of atherothrombotic disease. NGAL has performed as a more consistent predictor of adverse outcomes in patients with ACS, especially STEMI, compared with patients having stable CAD [132]. NGAL was associated with increased risk of long-term CHD events, independent of conventional risk factors and biomarkers [133].

## 6. MMPs as Biomarkers in Cardiovascular Diseases

MMPs are involved in various stages of plaque progression that are important to predict future atherothrombotic cardiovascular events [134]. Vulnerable atherosclerotic plaques are responsible for life-threatening clinical endpoints; thus, the best approach for both stroke and myocardial infarction (MI) would be safe diagnosis and knock out of the vulnerable lesion before endpoints occur [135]. Higher markers of matrix remodeling, such as MMP-9 and TIMP-1, were associated with a greater prevalence of carotid stenosis and subclinical atherosclerosis in the internal carotid artery (IC) [136]. Sequence variants at the MMP-1 genomic locus may influence risk of coronary heart disease in humans [137]. MMP-1 (-1607 1G/2G) and MMP-3 (-1171 5A/6A) polymorphisms may contribute to different subtypes of ischemic stroke susceptibility [138].

**Figure 3:**
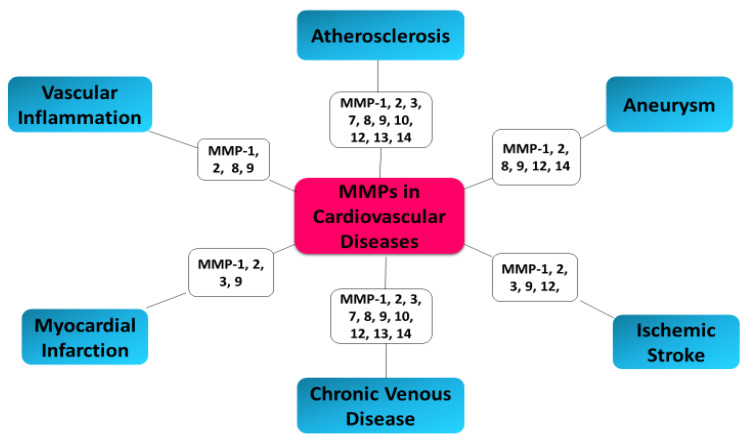
MMPs as biomarkers in cardiovascular diseases.

Since 2005, Sapienza and coauthors showed that an imbalance exists between MMPs and TIMPs in unstable carotid plaques, which is reflected in the plasma levels of these markers [139]. Disturbed equilibrium of the metalloproteinase/tissue inhibitors system, destabilization of atherosclerotic plaque, and acute coronary syndrome (ACS) in patients are caused by increased expression of MMP-2 and MMP-9 metalloproteinases and their tissue inhibitor (TIMP-2) [140]. Studies have indicated that MMP-2, MMP-8, and MMP-9 facilitate plaque rupture and clinical events [18,118]. In 2006, Turu et al. demonstrated significantly higher MMP-8 plasma concentrations in patients with unstable atherosclerotic plaques compared to patients with stable plaques [141]. The results of recent research confirm the relationship between MMP-8 and the processes of remodeling and destabilization of the plaque. A demonstrated relationship between the variability of the MMP-8 gene and atherosclerosis suggest that MMP-8 concentrations may have prognostic and diagnostic significance in assessing the patient’s cardiovascular risk [142]. MMP-8 has also been shown to be a proteinase responsible for the activation of other MMPs, such as MMP-2 and MMP-9 [143,144]. MMP-2 promotes a platelet aggregation, which is involved in a prothrombotic effect of atherosclerotic plaques. Enhanced MMP-2 activity was especially observed in plaques of patients with a higher rate of subsequent major cardiovascular events [145]. MMP-2 has been determined as a predictor of mortality in patients with ACS [146]. High levels of MMP-8 in the carotid plaque correlate with an unstable plaque composition and systemic cardiovascular outcome [147]. Seifert et al. confirmed that MMP-2 and MMP-9 activities was significantly higher in the ApoE-/- cuff model in unstable atherosclerotic plaques as compared to downstream more stable plaque phenotypes [148]. Increased plasma MMP-9 and TIMP-1 levels have been demonstrated in the coronary circulation in patients with acute coronary syndrome (ACS), which suggests active process of plaque rupture and future risk of cardiovascular events [149]. Kelly et al. have shown the association of MMP-9 and TIMP-1 with function and remodeling of left ventricular (LV) as well as cardiovascular death or heart failure [150]. The correlation between TIMP-1 and MMP-9 and echocardiographic parameters of LV remodeling after AMI (acute myocardial infarction) suggest a promising indicator of patients with adverse prognosis. In type 2 diabetes mellitus, elevated MMP-7 and MMP-12 plasma levels are linked with severe atherosclerosis as well as more frequent coronary events [151]. The stronger elevated plasma levels of MMP12 and imbalance between MMP12 and TIMP1 was observed in patients with STEMI compared to patients with stable angina pectoris [152]. Moreover, another study has shown elevated peripheral blood levels of MMP-2 and MMP-9 in patients with ACS [153]. Blankenberg et al. demonstrated that plasma MMP-9 concentrations may constitute risk markers for future cardiovascular death in ACS patients [154]. However, this study also has shown the correlation between MMP-9 level and acute-phase reactants; therefore, independent prognostic information of MMPs is not clear. Moreover, increased levels of TIMPs are associated with enhanced risk of cardiovascular events in patients with ACS [155,156]. Elevated MMPs levels have been shown not only in affected tissue in patients with ACS, arthritis, and cancer but also in peripheral blood, suggesting elevated blood MMPs levels in patients at risk of acute plaque disruption [153]. Higher plasma MMP-9 levels have been shown in patients with significant carotid stenosis undergoing carotid endarterectomy with detected spontaneous embolization, compared to patients without it [157]. MMP-9 expression and macrophage infiltration constitute strong indicators of high risk atherosclerotic carotid plaques and plaque instability [158,159]. MMP-9 may have vital significance in differentiating patients with unstable angina with or without plaques [160]. In patients with angiographically confirmed coronary heart disease (CHD), it was showed that increased concentrations of MMP-9 at baseline were associated with future cardiovascular (CV) death [161]. Tang et al. demonstrated that increased plasma levels of MMP2 and MMP9 in patients with coronary heart diseases (CHD) suggest the instability of the atherosclerotic plaque in correlation to the severity of ACS, and may serve as good indicators for the prediction of ACS and diagnosis of chronic total occlusion (CTO) of the coronary artery [162]. MMP-2 and MMP-9 circulating levels can serve as indicators of efficiency of the therapy provided to heart failure (HF) patients, as well as for identification of patients who could benefit from particular therapeutic intervention via modification of MMP pathway [163] (Figure 3).

## 7. Conclusions

Accumulating evidence confirm the key role of MMPs in plaque development and pathogenesis of atherosclerosis, especially in the advanced stages of the disease, where their elevated activity increases the risk of plaque rupture. The degradation of extracellular matrix protein is catalyzed by MMPs and participates in the migration of vascular SMCs and consequently, leads to plaque instability. Increased peripheral blood MMP-2 and MMP-9 in acute coronary syndrome (ACS) may be useful as noninvasive tests for detection of plaque vulnerability. Local carotid plaque MMP-8 level corresponds with a higher frequency of secondary manifestations of cardiovascular disease during follow-up, and increased plasma levels are predictive for subsequent all-cause mortality. Mentioned findings suggest that local MMPs plaque levels may be useful in detection of the vulnerable plaque and can help to predict patients with high risk of atherosclerotic cardiovascular events. The use of biomarkers to select patients for individualized therapies in secondary prevention will help achieve the goal of precision medicine. [164]. In addition, modulation of immune-mediated inflammation is a new promising point of action for the eradication of fatal atherosclerotic events [135]. The Canakinumab Anti-Inflammatory Thrombosis Outcome Study (CANTOS) trial confirmed that targeting inflammation with interleukin-1β (IL-1 β) inhibition significantly reduces cardiovascular events [165].

## Figures and Tables

**Table 1:** The family of matrix metalloproteinases (MMPs), tissue distribution, and substrates.

SUBFAMILY	MMP	Tissue Distribution	Substrates
**Collagenases**			
Collagenase-1	MMP-1	Endothelial cells, VSMCs, vascular wall, platelets, fibroblasts, macrophages	Collagens: I, II, III, VII, VIII, X Gelatins, entactin, aggrecan, link protein
Collagenase-2	MMP-8	Macrophages, neutrophils	Collagens: I, II, III; Aggrecan link protein
Collagenase-3	MMP-13	Vascular wall, SMCs, macrophages	Collagens: I, II, III
**Gelatinases**			
Gelatinase-A	MMP-2	Endothelial cells, VSMCs, adventitia, leukocytes, dermal fibroblasts, platelets	Collagens: I, IV, V, VII, X, XI; Gelatins, elastin, fibronectin, laminin, b-amyloid protein precursor
Gelatinase-B	MMP-9	Endothelial cells, VSMCs, adventitia, vascular wall, macrophages	Collagens: IV, V, XIV; Gelatins, elastin, entactin, vitronectin
**Stromelysins**			
Stromelysin-1	MMP-3	Endothelial cells, VSMCs, vascular wall, platelets	Collagens: III, IV, IX, X; Aggrecan, fibronectin, laminin, elastin, gelatins, casein
Stromelysin-2	MMP-10	Uterus	Collagens: II, IV, V; Aggrecan, fibronectin, gelatins, activate procollagenase
Stromelysin-3	MMP-11	Uterus, brain	Collagen IV, weakly fibronectin, laminin, aggrecan, gelatins, IGFBP-1, a1-protease inhibitor
**Matrilysins**			
Matrilysin-1	MMP-7	Endothelial cells, VSMCs, vascular wall, uterus	Collagen IV, aggrecan fibronectin, laminin, entactin, vitronectin, casein, IGFBP-1
Matrilysin-2	MMP-26		Collagen IV, gelatin, fibronectin
**Membrane-type MMPs**			
MT1-MMP	MMP-14	Vascular wall, platelets, fibroblasts, uterus, brain	Collagens I, II, III; fibronectin, laminin-1, dermatan sulfate
MT2-MMP	MMP-15	Fibroblasts, leukocytes	Large tenascin-C, fibronectin, laminin, entactin, aggrecan, perlecan
MT3-MMP	MMP-16	Vascular wall, leukocytes	Collagen III, gelatin, casein, fibronectin
MT4-MMP	MMP-17	Brain	Activates MMP2 by cleavage
MT5-MMP	MMP-24	Leukocytes, lung, pancreas, kidney, brain	Activates MMP2 by cleavage
MT6-MMP	MMP-25	Leukocytes	Inactivates alpha-1 proteinase inhibitor
**Other**			
Metalloelastase	MMP-12	VSMCs, fibroblasts, macrophages, great saphenous vein	Elastin, fibronectin
RASI-1	MMP-19	Liver	Gelatin
Enamelysin	MMP-20	Tooth enamel	Amelogenin
Xenopus-MMP	MMP-21	Fibroblasts, macrophages, placenta	
CA-MMP	MMP-23	Ovary, testis	Gelatin
Epilysin	MMP-28	Skin, keratinocytes	Casein

**Table 2:** Participation of MMPs in processes involved in development and progression of atherosclerotic plaque.

**Vascular Inflammation and Endothelial Dysfunction**	
Increase of inflammatory cell migration and invasion into the arterial wall	MMP-1, MMP-8, MMP-9, MMP-12, MMP-13, MMP-14
Influence on endothelial dysfunction	MMP-2, MMP-9
Participation in oxLDL effect	MMP-1, MMP-2, MMP-8, MMP-9
Promotion of EC apoptosis	MMP-9
**Development of Plaque**	
Increase of intima–media thickness	MMP-1, MMP-2, MMP-3, MMP-7, MMP-8, MMP-9, MMP-10
Promotion of plaque growth	MMP-2
Promotion of VSCMs migration	MMP-9
Decrease of VSCMs contractility	MMP-2, MMP-9
**Neovascularization**	
Degradation of ECM	MMP-1, MMP-8, MMP-9, MMP-12, MMP-13, MMP-14
Release of growth factors	MMP-2, MMP-9, MMP-13, MMP-14
**Calcification**	
Provoke vascular calcification	MMP-2, MMP-7
**Plaque Activation and Destabilization**	
Apoptosis of VSMCs in the fibrous cap	MMP-7
Collagenolytic activity	MMP-1, MMP-8, MMP-13
Enhances plaque rupture	MMP-1, MMP-2, MMP-3, MMP-7, MMP-8, MMP-9, MMP-12, MMP-13, MMP-14
